# Impact of short-term extreme temperature events on physiological performance of *Salicornia ramosissima* J. Woods under optimal and sub-optimal saline conditions

**DOI:** 10.1038/s41598-018-37346-4

**Published:** 2019-01-24

**Authors:** Jesús Alberto Pérez-Romero, Jose-Maria Barcia-Piedras, Susana Redondo-Gómez, Enrique Mateos-Naranjo

**Affiliations:** 1 Departamento de Biología Vegetal y Ecología, Facultad de Biología, Universidad de Sevilla, 1095, 41080 Sevilla, Spain; 2 Department of Ecological Production and Natural Resources Center IFAPA Las Torres-Tomejil Road Sevilla - Cazalla Km 12′2, 41200 - Alcalá del Río, Seville, Spain

## Abstract

Increasing extreme temperature climatic events could exert an important effect on plant photosynthetic performance, which could be modulated by the co-occurrence with other environmental factors, such as salinity, in estuarine ecosystems. Therefore, a mesocosm experiment was designed to assess the impact of temperature events for three days (13/5 °C, 25/13 °C and 40/28 °C) in combination with two NaCl concentrations (171 and 1050 mM NaCl) on the physiological performance of *Salicornia ramosissima*. Extreme temperature events had a negative impact on *S*. *ramosissima* photosynthetic efficiency, this effect being more marked with cold wave at both salinities, compared with heat wave, even in presence of NaCl excess. This differential thermotolerance in the photosynthetic apparatus was ascribed to the greater integrity and functioning of its photosynthetic pathway at high temperature, as indicated by constant g_s,_ *V*_c,max_ values at optimal salinity and the higher values of those parameters and g_m_ recorded in combination with NaCl excess. Moreover, *S*. *ramosissima* was able to upregulate the energy sink capacity of its photochemical apparatus at elevated temperature and salinity by a greater energy excess dissipation capacity. This could have contributed to reducing the risk of oxidative stress, along with the recorded higher capacity for antioxidant enzyme activity modulation under these conditions.

## Introduction

Together with the recognized atmospheric CO_2_ enrichment and temperature increment, climate change models indicate that extreme climatic events, such as salinization, floods, drought and cold and heat waves are likely to increase, not only in intensity but also in frequency, especially in the Mediterranean area^1^. To date, there have been increasing reports investigating the effect of the main slower environmental changes related to climate change, such as atmospheric CO_2_ enrichment or temperature increment, on plants species composition, structure and distribution^2^. In spite of this increasing number of reports, not many studies have addressed the impact of short extreme climatic events, such as heat and cold waves on plant species, despite the fact that it has been stated that in many cases these phenomena might have an important selective effect on plant species, limiting its development and distribution by dysfunction of essential biological processes such a plant phenology, reproduction, physiology, etc.^3^. Furthermore, the few existing studies only assessed the impact of a single extreme climatic event, not taking into account the co-occurrence with other important environmental factors that are likely to limit species survival, development and distribution. This is the case of estuarine ecosystems, where plant species survival and distribution is highly modulated by stressful abiotic factors such salinity, flooding, redox potential, etc.^4^. Therefore, assessing the response of these plants species to extreme thermal climate events, in co-occurrence with medium salinity level variations, is of paramount importance for understanding the future fitness of these species. This experiment was thus designed and conducted to fill these gaps in knowledge.

*Salicornia ramosissima* J. Woods (Chenopodiaceae) is a C_3_ species which has been considered an alternative multifunctional cash crop for many arid and semiarid regions of the world^5^, due to its edibility^6^ and its high physiological versatility, which allows it to tolerate a wide range of environmental factors, pollution^7^, salinity^8^, etc. Recently, like for other halophytes species^8–13^, some authors have emphasized the positive impact of rising atmospheric CO_2_ linked with climate change on *S*. *ramosissima* performance under sub-optimal salinity conditions^8^. Accordingly, we hypothesize that extreme short climatic events, such cold and heat waves, would lead to differential photochemical responses in *S*. *ramosissima* under optimal and suboptimal salinity conditions, with the consequent effect on the primary productivity and development of this important multifunctional cash crop species. Therefore, this study aimed to: (1) ascertain the extent to which photosynthetic apparatus (PSII chemistry), gas exchange characteristics (CO_2_ diffusive and biochemical component) and photosynthetic pigments profiles determine plant tolerance to cold (13/5 °C) and heat (40/28 °C) waves under optimal (171 mM NaCl) and suboptimal saline conditions (1050 mM NaCl); and (2) examine possible role of antioxidant enzyme machinery in these responses.

## Results

### Osmotic potential and water content measurements

**Figure 1:**
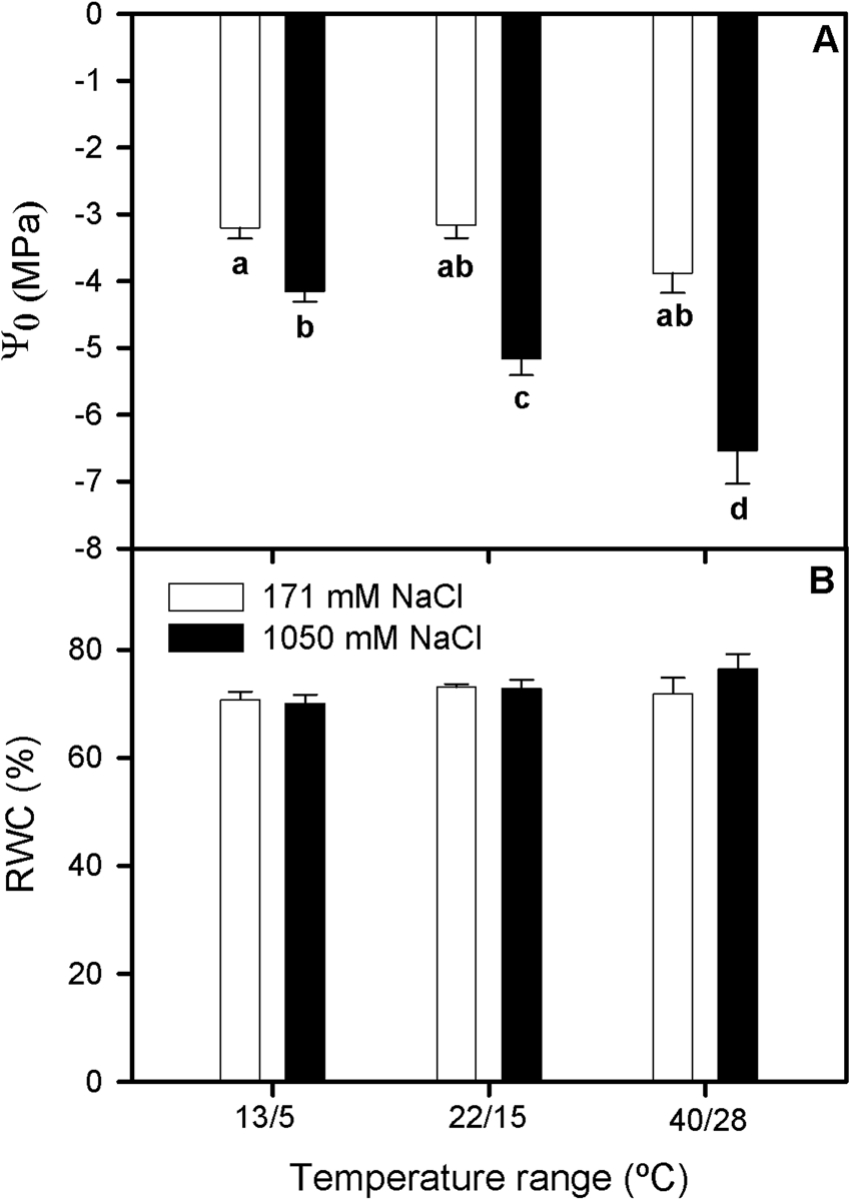
Osmotic potential, Ψ_o_ (**A**) and relative water content, RWC (**B**), in randomly selected, primary branches of *Salicornia ramosissima* in response to treatment with three temperature ranges (13/5 °C, 22/15 °C and 40/28 °C) and two NaCl concentrations (171 and 1050 mM) after 3 days. Values represent mean ± SE, n = 10. Different letters indicate means that are significantly different from each other.

Overall, Ψ_o_ values increased in plants grown at 1050 mM NaCl compared with their non-high salinity treated counterparts, this increment being more marked in those exposed to 40–28 °C (Two-way ANOVA_NaCl_, F_1,42_ = 70.123; Fig. 1A). Contrarily, there were not significant effects of salinity and temperature ranges treatments on RWC, in all cases the values being between 75–80% (Fig. 1B).

### Gas exchange measurements and photosynthesis limitation analysis

**Figure 2:**
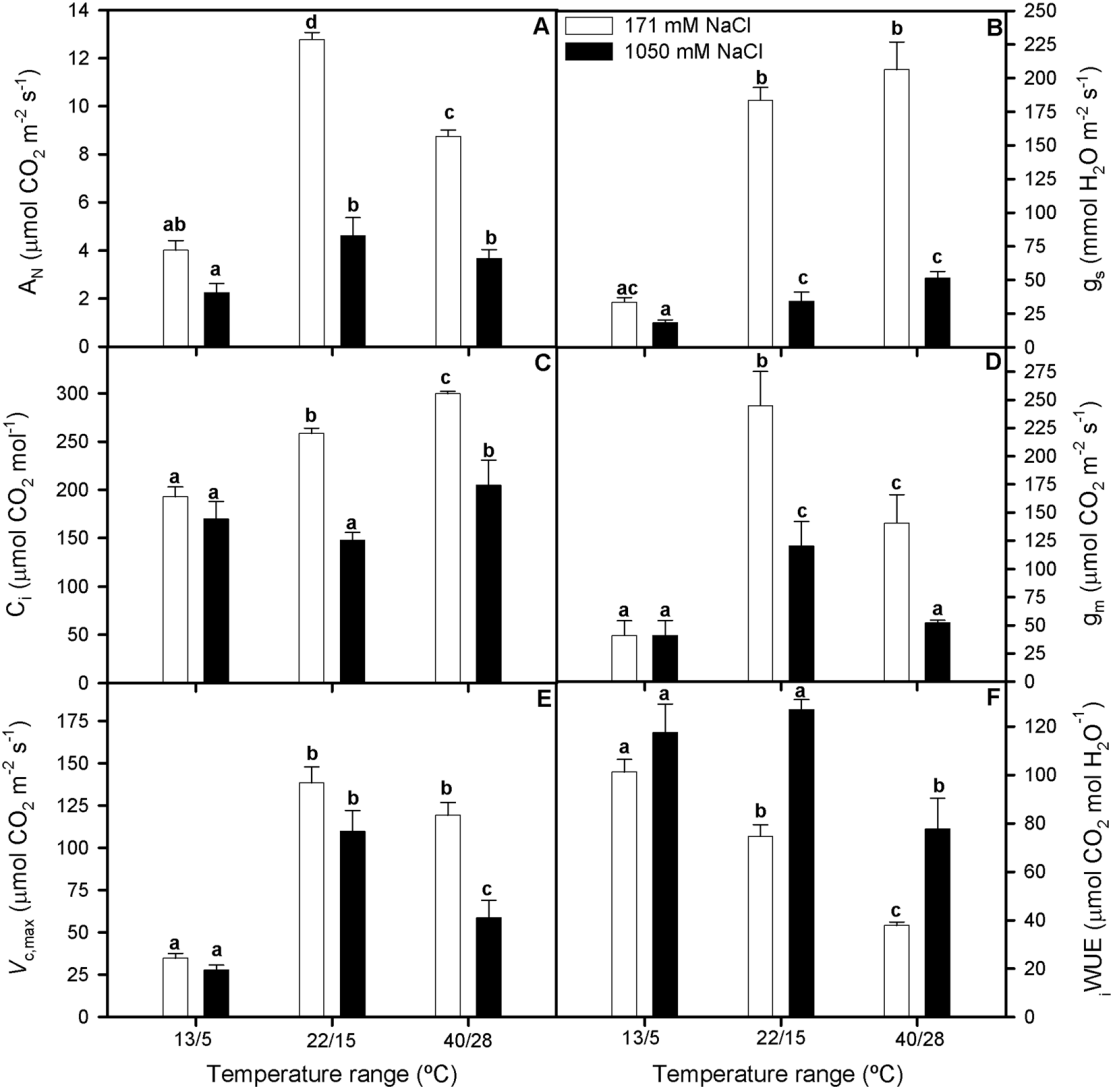
Net photosynthetic rate, A_N_ (**A**), stomatal conductance, g_s_ (**B**), intercellular CO_2_ concentration, C_i_ (**C**), mesophyll conductance, g_m_ (**D**), maximum carboxylation rate, *V*_c,max_ (**E**) and intrinsic water use efficiency (_i_WUE) (**F**) in randomly selected, primary branches of *Salicornia ramosissima* in response to treatment with three temperature ranges (13/5 °C, 22/15 °C and 40/28 °C) and two NaCl concentrations (171 and 1050 mM) after 3 days. Values represent mean ± SE, n = 10 for A_N_, g_s_, C_i_ and _i_WUE and n = 4 for g_m_ and *V*_c,max_. Different letters indicate means that are significantly different from each other.

There were significant effects of both salinity and temperature range treatments on the gas exchange characteristics of *S*. *ramosissima* after 3 d of treatment (Two-way ANOVA_NaCl_, F_1,138_ = 33.333; Two-way ANOVA_T_ F_1,132_ = 21.578; Fig. 2A–F). Thus, A_N_ values decreased considerably by NaCl excess in the grown medium and in plants grown at both extreme temperature ranges respect to the control, this temperature effect being more marked at low-temperature range and with salinity excess at both temperature range treatments. Hence, compared to the control (i.e medium temperature range and optimum salinity concentration) A_N_ decreased 31% and 71% in plants grown at 171 mM NaCl and temperature range of 40/28 °C and 13/5 °C, respectively; while at 1050 mM NaCl these reductions were of 71% and 82% for those temperature ranges. Very similar trends were recorded for g_s,_ g_m,_ *V*_c,max_ and C_i_ but high-temperature range treatment did not affect g_s,_ *V*_c,max_ and C_i_ in plant grown at 171 mM NaCl. In addition, *V*_c,max_ values were not affected by NaCl excess in plants grown at 25/13 °C and by high-temperature range in those grown at 171 mM NaCl compared with their non-high salinity and temperature treated counterparts (Fig. 2E). Contrarily, _i_WUE values were higher in plant expose to 1050 mM NaCl and decreased with temperature range increment (Fig. 2F).

**Figure 3:**
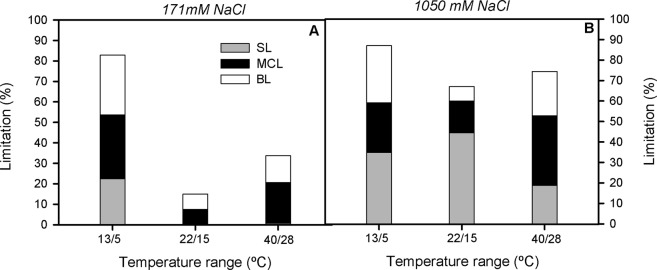
Quantitative limitation of photosynthesis in randomly selected, primary branches of *Salicornia ramosissima* in response to treatment with three temperature ranges (13/5 °C, 22/15 °C and 40/28 °C) and with 171 mM NaCl (**A**) and 1050 mM NaCl (**B**) after 3 days. SL, MCL and BL denote for stomatal, mesophyll and biochemical limitations, respectively. Values represent mean ± SE, n = 4.

Regarding the photosynthesis quantitative limitation analysis (Fig. 3A,B), indicated that the observed decreases of A_N_ after 3 d of treatment, were mainly due to diffusional limitations, although the relative importance of stomatal (SL) and mesophyll conductance limitation (MCL) varied depending of medium salinity concentration and temperature range treatment. Thus, SL accounted for the highest percentage of photosynthetic limitation under sub-optimal salinity concentration (e.i. 1050 mM NaCl) in plants grown at low and medium temperature ranges, but the relative importance of MCL was higher in plants grown at 40/28 °C in both salinity concentrations and in those subjected to 13/5 °C and 171 mM NaCl. In addition, there is to notice that biochemical limitation (BL) augmented in both extreme temperature ranges, this increment being more marked at 13/5 °C, regardless of the medium NaCl concentration.

### Fluorescence measurements

**Figure 4:**
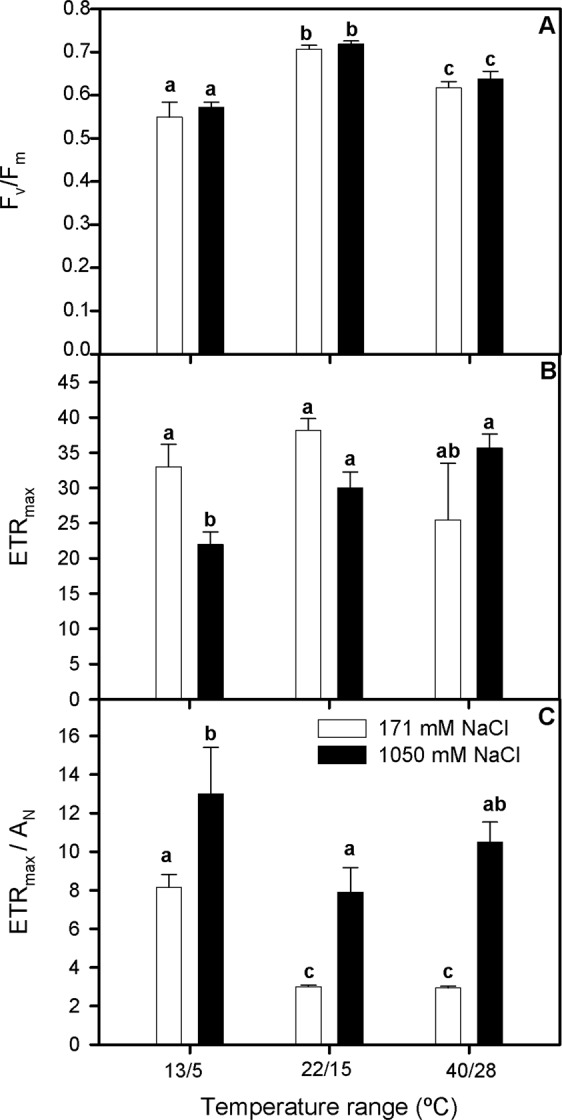
Maximum quantum efficiency of PSII photochemistry, F_v_/F_m_ (**A**), maximum ETR after which photo-inhibition can be observed, ETR_max_ (**B**) and ETR_max_/A_N_ (**C**) in randomly selected, primary branches of *Salicornia ramosissima* in response to treatment with three temperature ranges (13–5 °C, 22–15 °C and 40–28 °C) and two NaCl concentrations (171 and 1050 mM) after 3 days. Values represent mean ± SE, n = 7. Different letters indicate means that are significantly different from each other.

Chlorophyll fluorescence parameters were also affected by salinity and temperature ranges treatments after 3 d. F_v_/F_m_ values decreased at both low and high temperatures ranges, this reduction being more marked at low-temperature range but without significant differences between salinity treatments (Two-way ANOVA_T_, F_1,64_ = 21.393; Fig. 4A). Contrarily, ETR_max_ did not remarkably vary between salinity and temperature ranges treatments, except in plants grown at 1050 mM NaCl and 13/5 °C, which showed the lowest values (Two-way ANOVA_NaClxT_, F = 5.554; Fig. 4B). As a consequence, ETR_max_/A_N_ values were higher in plants grown at 1050 mM NaCl compared with their non-NaCl supplied counterparts in all temperature ranges treatments, and tended to increase in plants grown at 13/5 °C in both salinity concentration treatments (Fig. 4C).

**Figure 5:**
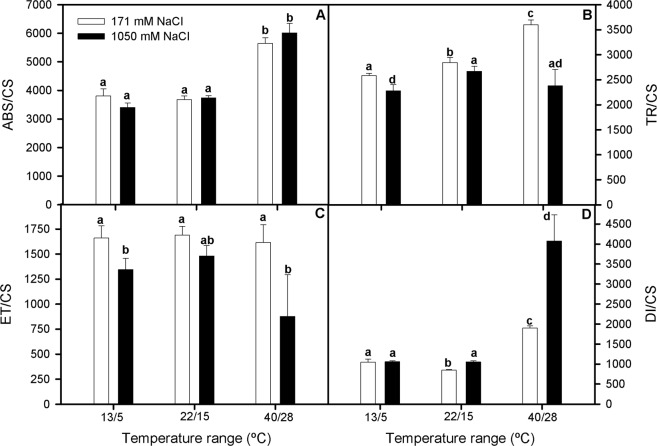
Absorbed energy flux, ABS/CS (**A**), trapped energy flux, TR/CS (**B**), transport energy flux ET/CS (**C**) and dissipated energy fluxes, DI/CS (**D**) per cross section in randomly selected, primary branches of *Salicornia ramosissima* in response to treatment with three temperature ranges (13/5 °C, 22/15 °C and 40/28 °C) and two NaCl concentrations (171 and 1050 mM) after 3 days. Values represent mean ± SE, n = 7. Different letters indicate means that are significantly different from each other.

Focusing on the energetic fluxes on a leaf cross-section basis (phenomological fluxes), since reaction centres per cross section (RC/CS) varied between salinity and temperature range treatments (data not presented), our results showed that absorbed energy flux per leaf-cross section (ABS/CS) was significantly higher at high-temperature range, with no significant differences between NaCl treatments (Two-way ANOVA_T_, F_1,54_ = 37.892; Fig. 5A). Moreover, trapped energy flux (TR/CS) values increased at high-temperature range and 171 mM NaCl, together with dissipated energy flux per cross section (DI/CS) values in plants grown at both salinity treatments. This effect, however, was more marked at 1050 mM NaCl (Two-way ANOVA_NaClxT_, F_2,36_ = 14.654; Fig. 5B,D). Finally, electron transport energy per leaf cross section (ET/CS) did not vary with salinity and temperature treatments except in plants grown at 1050 mM NaCl and 40/28 °C, which showed the lowest values (Two-way ANOVA_NaClxT_, F_2,54_ = 3.243; Fig. 5C).

### Photosynthetic pigment concentration

Chlorophyll *a* and *b* concentrations did not vary between salinity and temperature ranges treatments, except in plants grown at 1050 mM and 40/28 °C, for which the lowest values were recorded, compared with the other treatments (Two-way ANOVA_NaClxT_, F_2,24_ = 24.573, F_2,24_ = 16.104; Table 1). Very similar trends were recorded in pheophytine *a*, β-carotene, neoxanthin and zeaxanthin concentrations; while lutein concentration did not vary between experimental treatments and violaxanthin concentration was significantly lower in plants grown at 13/5 °C independently of salinity treatment (Table 1). Finally, the greatest DES values were recorded in plants grown at high-temperature range and 1050 mM (Table 1).

**Table 1:** Photosynthetic pigments concentrations (µg /g) and deposidation state in randomly selected branches of *Salicornia ramosissima* in response to treatment with three ranges of temperatures and two NaCl concentrations (171 and 1050 mM) after 3 days. Values represent mean ± SE, n = 5. Different letters indicate means that are significantly different from each other.

T (°C)	[NaCl]	Chl *a*	Chl *b*	Phe *a*	β-carotene	Lutein	Neoxanthin	Violaxanthin	Zeaxanthin	DES
13/5	171	111.1 ± 6.5^a^	82.7 ± 6.0^a^	156.6 ± 20.9^ab^	8.6 ± 1.7^ab^	17.1 ± 1.4	13.5 ± 1.7^ab^	0.7 ± 0.6^b^	9.1 ± 1.8^ab^	0.04 ± 0.04^c^
	1050	119.5 ± 9.7^a^	84.0 ± 5.0^a^	124.2 ± 8.6^ab^	7.1 ± 0.6^ab^	15.1 ± 1.5	9.6 ± 1.5^b^	0.8 ± 0.5^b^	7.5 ± 0.6^ab^	0.21 ± 0.07^a^
22/15	171	168.1 ± 31.7^a^	92.7 ± 9.9^a^	176.7 ± 20.9^a^	11.6 ± 1.8^a^	16.2 ± 4.0	13.9 ± 1.2^ab^	3.4 ± 2.2^a^	12.3 ± 1.9^ab^	0.13 ± 0.09^a^
	1050	125.4 ± 10.1^a^	88.4 ± 9.3^a^	156.9 ± 19.4^ab^	10.9 ± 1.7^a^	15.3 ± 1.5	15.1 ± 1.4^ab^	2.1 ± 1.1^a^	11.6 ± 1.8^ab^	0.16 ± 0.05^a^
40/28	171	166.9 ± 43.8^a^	104.2 ± 19.7^a^	137.1 ± 24.7^b^	11.4 ± 2.9^a^	21.7 ± 5.2	18.2 ± 7.0^a^	2.6 ± 0.7^a^	12.1 ± 3.1^a^	0.19 ± 0.07^a^
	1050	61.1 ± 4.4^c^	51.1 ± 5.3^b^	72.4 ± 11.5^c^	5.5 ± 0.7^b^	14.9 ± 2.4	8.5 ± 1.8^b^	2.7 ± 0.9^a^	5.9 ± 0.7^b^	0.39 ± 0.00^b^

### Anti-oxidant enzymatic activity measurements

**Figure 6:**
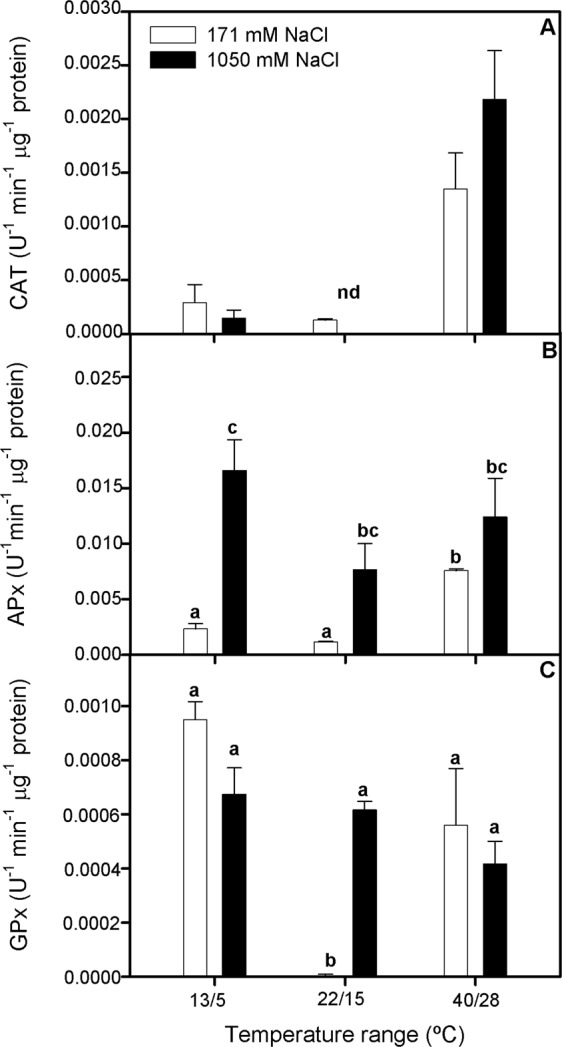
Catalase, CAT (**A**), ascorbate peroxidase, APx (**B**) and guaiacol peroxidase, GPx (**C**) activity in randomly selected, primary branches of *Salicornia ramosissima* in response to treatment with three temperature ranges (13/5 °C, 22/15 °C and 40/28 °C) and two NaCl concentrations (171 and 1050 mM) after 3 days. Values represent mean ± SE, n = 3. Different letters indicate means that are significantly different from each other.

CAT activity tended to increase in plants grown at high-temperature range but without significant differences between experimental treatments (Fig. 6A). While APx activity was overall higher in plants exposed to additional NaCl supplementation for all temperature range treatments, except at 40/28 °C, where this activity also increased in plants grown at 171 mM NaCl (Two-way ANOVA_NaClxT_, F_2,14_ = 12.675; Fig. 6B). Finally, GPx enzyme activity was invariably high for all salinity and temperature treatments compared to the optimum grown conditions (i.e. medium temperature range and optimum salinity concentration; Fig. 6C).

## Discussion

Increasing frequency and magnitude of short extreme thermal climatic events resulting from anthropogenic activities^1^ will induce loss of carbon assimilation^14^, with the consequent deleterious effect on the development and survival of many plant species. However, it has been described that plant species could present differential thermotolerance or sensitivity to cold or heat induced stress, these responses being species specific and dependent on the thermal characteristics of the niche they inhabit. The development of particular adaptation mechanisms could also allow them to cope with these extreme thermal conditions^15^. In this study we have explored the ways in which different short extreme thermal events could affect photosynthesis, the basis of plant bio-chemical system, in the halophytic species *S*. *ramosissima*. In addition, we have assessed if and how this impact could be modulated by the co-ocurrence of other stressful factors, such as the characteristic salt excess present in the original habitat of this species.

Our study showed that extreme temperature events had a negative impact on *S*. *ramosissima* photosynthetic performance after 3 days of exposition, the impairment degree temperature range treatment-dependent and modulated by the NaCl concentration in the growth medium only in plants grown at the heat-wave treatment. Thus, cold wave limited to a greater extent CO_2_ assimilation capacity of *S*. *ramosissima* regardless of medium saline concentration compared with heat-wave treatment even in presence of NaCl excess. Moreover, the results showed that salinity excess exacerbated the negative impact of heat wave on the photosynthetic performance of *S*. *ramosissima*. However, this differential temperature and salinity deleterious effect were not reflected in *S*. *ramosissima* water imbalance, as indicated by the invariable RWC values due to changes in Ψ_0_ and _i_WUE in response to salinity and temperature excess.

One of the main reasons for this better photosynthetic efficiency of *S*. *ramosissima* under heat stress with both optimal and sub-optimal salinity was related to the greater integrity and functioning of some steps of its photosynthetic pathway, namely stomatal and mesophyll diffusion behavior, Rusbisco activity and light-harvesting antenna efficiency to temperature excess. Hence, although photosynthetic injury as a result of both tested extreme temperature ranges was mainly due to CO_2_ diffusion limitation, the degree and the relative importance of the stomatal and mesophyll component differed in each specific temperature and salinity combination treatment, as our photosynthetic limitation analysis also indicated. Thus, cold wave imposed a sharply decrease in both g_s_ and g_m_ regardless of salinity treatment. This similar diffusion limitation when cold and salinity stress act individually or in combination suggests that the primary effects of low temperature and salinity excess are similar and not synergistic. Accordingly, similar g_s_ and g_m_ decreases have been observed in many sensitive species to low temperature and saline excess as a single factor, this response being linked with an ameliorated water stress induced by these stresses^16,17^. Contrarily, heat-wave treatment only increased stomatal limitation to CO_2_ diffusion under hypersaline conditions, diffusional limitation to CO_2_ appearing as a major result of g_m_ reduction under optimal saline. The absence of heat-wave effect on g_s_ could be associated with regulatory mechanisms to enhance leaf cooling through transpiration^18^. Regarding the effect of salinity excess, Redondo-Gómez *et al*.^19^ also recorded a marked reduction in g_s_ of *Sarcocornia fruticosa* grown at 1030 mM NaCl. The observed trends in g_s_ and g_m_ resulted in an increase in C_i_ in plants grown at optimum salinity, where CO_2_ was being transported to the intercellular space. However, it was not transferred to chloroplast due to a reduction in g_m_ with the consequent accumulation. Therefore, our results revealed a lack of relationship between g_s_ and g_m_ in *S*. *ramosissima* in response to heat wave and medium salinity concentration. Many studies suggest that g_s_ and g_m_ are co-regulated, since both variables respond in a similar way and magnitude to several environmental factors^20^. Nonetheless, like in our experiment, some authors have identified differential responses in both parameters under severe stress situations^21,22^. Moreover, although there is not a general consensus, it seems that the existence or not of a possible relationship between both parameters would be highly influenced by specific environmental variables as well as by the co-existence with other environmental factors^23^. This response would allow *S*. *ramosissima* to have a lower diffusional limitation when coping with heat waves. Furthermore, extreme temperature events could head to biochemical limitations of photosynthetic process due to inhibition or modifying carboxylase activity of Rubisco^24–26^. Hence, there are records of a reduction in Rubisco carboxylation rate at all temperatures below 25 °C and in particular at 5 °C, mainly on account of impairment in the activation state^24^. A very similar injury effect on Rubisco kinetics has also been reflected in plants when temperature exceeds the optimum values for the photosynthesis process^14,26^. Our results indicated that photosynthesis biochemical limitation in *S*. *ramosissima* augmented in both extreme temperature ranges but in greater proportion in cold-temperature range treatment at both salinity concentrations. Thus, it is noteworthy that *V*_c,max_ values did not vary respect to the control (i.e medium temperature range and optimum salinity concentration) in plants exposed to 1050 mM NaCl and 22/15 °C, as well as in those subjected to heat wave and optimum salinity conditions. This result suggested that the high tolerance of *S*. *ramosissima* to heat waves even under salinity excess would be in part explained by the maintenance of a high Rubisco activation rate. Contrarily, Perdomo *et al*.^26^. found an increment of biochemical limitation at high temperature mainly attributed to a decrease in Rubisco activation state. However, Cen and Sage^27^ indicated that, although temperature increment led to deactivating Rubisco activase, this deactivation was not sufficient to limit photosynthesis. In fact, it has been described that several iso-forms of Rubisco activase could play a role in the responses of plant tolerant to high temperature stress^14^. Accordingly, it is possible that the presence of Rubisco activase iso-forms that are highly tolerant to heat and salinity induced stress in *S*. *ramosissima*. This area is therefore worthy of further research.

On the other hand, the higher photosynthetic thermotolerance of *S*. *ramosissima* to elevated temperature range could be ascribed to the upregulation of the energy sink capacity of its photochemical apparatus, as well as to the impact on the photosynthetic pigments profile. In this regard, one of the best recognized effects of high temperature is the destruction of PSII components; however, PSII damage in well light-adapted systems, as could be the case of *S*. *ramosissima*, is supposed to occur only at very high temperatures (i.e. >45 °C^28^). Accordingly, our results indicated that, although photochemical efficiency (F_v_/F_m_) of *S*. *ramosissima* was significantly lower at both extreme temperature ranges, this effect was more drastic at the cold-temperature treatment. This trend was also confirmed by the lower ETR_max_ values recorded, especially in plants grown at 13/5 °C and 1050 mM NaCl. In addition, OJIP analysis provided a downscaling approach to this highlighted differences in the thermotolerance behavior of *S*. *ramosissima*. Thus, when energy fluxes were assessed, we found that plants treated under high-temperature range showed greater values for ABS/CS and TR/CS compared with the other treatments. This fact was related to higher values obtained for these plants in the connectivity of their PSII units and the lower values of closure rate for its RC, respectively^29^. This could indicate that more energy was being absorbed and transformed in their photosystem. In addition, an increment of ABS/CS means a greater number of active reaction centers functioning as a heat radiator, protecting the plant against high temperature and light intensities^29^. This was also associated to an increase in DI/CS, especially at 1050 mM NaCl, which could explain the lower values for ET/CS obtained and indicates the activation of some defense mechanism such as the dissipation of energy as heat or photorespiration^30^. High salinity increased DI/CS, as Perez-Romero *et al*.^8^ found, and this was shown at high and control temperature range. Nonetheless, plants at low temperature did not show a significant increment in this parameter. The DI/CS values include non-photochemical quenching (NPQ), which is correlated with the xantophyll cycle that is activated under an excess of light^31^. The results obtained for the de-epoxidation of the zeaxanthin, showed as DES, suggested that there was an activation of this cycle at high-temperature range and hypersalinity. This data could be an evidence of a response to reactive oxygen species (ROS) stress for plants under these treatments of high temperature and salinity. Hence, this mechanism could help plants subjected to heat waves to reduce its physiological stress as ROS accumulation by dissipation of energy excess. Consequently, plants subjected to low temperature range and 171 mM NaCl showed the lowest values of this parameter indicating that this mechanism could be triggered by heat and not by cold, although previous studies suggested that neither heat nor cold waves induced this cycle^32,33^. A good approximation to knowing the potential risk of stress due to ROS is the ratio ETR_max_/A_N_, which could indicate the number of electrons that are not being fixed, so that they are free to possibly create some ROS^34,35^. This parameter indicated that high salinity significantly increases the risk of ROS stress in *S*. *ramosissima*. In addition, plants subjected to cold waves presented the highest values at both salinities, this fact corroborating the idea that cold waves have a greater injury effect on *S*. *ramosissima* physiological performance. Since cold and heat stress events can lead to an increment in ROS production^36,37^ there would be a need for antioxidative protection. One of the well-known mechanisms to reduce the accumulation of ROS is the modulation of the activity of antioxidative stress enzymes^9^. CAT and peroxidases as GPx and APx are enzymes that reduce the H_2_O_2_ produced^10^. Our experiment showed that antioxidant enzyme activity was modulated to a certain extent by temperature range characteristics and salinity excess, as indicated by the overall higher activation of GPx activity at both temperature range and salinity treatments compared with the control treatment. In addition, APx was also more activated for all temperature range treatments at 1050 mM NaCl than at 171 mM NaCl, as previously described for this species^38^. It is important to highlight that plants at both salinities subjected to heat wave showed higher activities for CAT and APx enzymes emphasizing the idea of higher capacity for antioxidant enzyme activity modulation of *S*. *ramosissima* in response to heat than to cold stress.

Thus, we can conclude that *S*. *ramosissima* photosynthetic metabolism would maintain its functionality better under heat waves than under cold waves, even in presence of hypersaline conditions during its vegetative natural cycle (i.e. from March to September^39^). This is mainly due to an improvement in its energy sink capacity while keeping its CO_2_ assimilation capacity relatively constant by ameliorating the diffusion and biochemical photosynthesis limitations under those extreme conditions. This pattern of response fits well with its thermal niche characteristics in the southwest of Iberian Peninsula, where there is a prevalence of daily mean high temperatures and a higher occurrence of heat waves, even at the beginning of its growth period. With cold waves tending to decrease in the Iberian Peninsula^40^, the higher photosynthetic efficiency of *S*. *ramosissima* against heat waves, even under hypersaline conditions could provide a great adaptation to these common conditions in its natural distribution area. However, the increase in frequency of extreme cold episodes due to climate change would compromise the primary productivity of this important multifunctional cash crop species.

## Material and Methods

### Plant Material

In September 2014 seeds of *S*. *ramosissima* were collected from Odiel marshes (37°15′N, 6°58′O; SW Spain) from a well-established population located in a well-drained intertidal lagoon (mean sea level +1.65 m relative to SHZ). Seeds were stripped for each plant and stored in the dark for 4 months at 4 °C until the beginning of the experiment.

In February of 2015 seeds were germinated on 9 cm Petri dishes filled with agar 10% into a germinator (ASL Aparatos Científicos M-92004, Madrid, Spain) with a day-night regime of 16 h of light (photon flux rate, 400–700 nm, 35 μmol m^−2^ s^−1^) at 25 °C and 8 h of darkness at 12 °C, for 15 days. The seedlings obtained were carefully transferred with thin tweezers from the agar to individual plastic pots (9 cm high × 11 cm diameter) using perlite as substrate and making sure that the perlite was tight enough to conduct the nutrient solution until the roots. These pots were placed in a greenhouse with controlled conditions (temperature between 21–25 °C, 40–60% relative humidity and natural daylight of 250 μmol m^−2^ s^−1^ as minimum and 1000 μmol m^−2^ s^−1^ as maximum light flux). Pots were allocated in shallow trays watering with 20% Hoagland’s solution^41^ and 171 mM NaCl. Plants were kept under these conditions until the experimental setup.

### Experimental treatments

In May of 2015, after 3 months of seedling culture, plants with an initial height of 17 cm were randomly divided in six blocks of 10 plants. They were then subjected to three different maximum and minimum temperature ranges (cold wave: 13/5 °C, control: 25/13 °C and heat wave: 40/28 °C) in combination with two NaCl concentrations (171 and 1050 mM NaCl) in controlled-environment chambers (Aralab/Fitoclima 18.000EH, Lisbon, Portugal) for 3 days after 2 days of salinity adaptation to emulate the effect of cold or heat waves. The salinity concentration of 171 mM NaCl was chosen as the optimum for *S*. *ramosissima* development, as we have stated previously^7^, and the higher one (e.i. 1050 mM NaCl) was added to reproduce temporarily hypersaline conditions in the Gulf of Cadiz marshes, as occurs in saltpans^42^. Low and high temperature range treatments were based on extreme events recorder recently in the southwest of Iberian Peninsula in the seasons when *S*. *ramosissima* presents its vegetative phase (aemet.es). These extreme salinity and temperature situations being likely to become more frequent in the future climatic reality^1^. Salinity treatments were established by using 20% Hoagland’s solution^41^ and NaCl of the appropriate concentration. Climatic chambers were programmed with alternating diurnal regime of 14 h of light and 10 h of darkness, light intensity of 1000 μmol m^−2^ s^−1^ and 40–60% relative humidity. During the experiment salinity concentrations were monitored and maintained at the same level.

Measurements of plant water status, gas exchange, chlorophyll fluorescence, photosynthetic pigments concentrations and anti-oxidant enzymatic activity were taken 3 days after the onset of temperature ranges treatments. All measurements, except for anti-oxidant enzymatic activity due to need for a greater amount of plant material, were made on fully developed internodes of similar size primary branches close to distal ends of principal stem, in order to facilitate the comparison between them and avoid the intra-plant modular variation in response to abiotic stress^43^.

### Measurement of osmotic potential and water content

At the end of the experiment, the osmotic potential (Ψ_o_) of primary branches (n = 10) was determined, using psychrometric technique with a Vapor Pressure Osmometer (5600 Vapro, Wescor, Logan, USA).

In addition, the relative water content (RWC) of primary branches (n = 10) in each treatment was calculated as follow:

$${\rm{RWC}}=(({\rm{FW}}-{\rm{DW}})/({\rm{FW}}-{\rm{TW}})){\rm{100}}$$

Where FW = fresh weight of the branches, DW = dry weight after oven-drying at 80 °C for 48 h and TW = turgid weight after being in water at 4 °C for 24 h.

### Measurement of gas exchange and photosynthetic limitation analysis

Gas exchange measurements were taken on randomly selected primary branches using an infrared gas analyser in an open system (LI-6400XT, LI-COR Inc., Neb., USA) equipped with a light leaf chamber (Li-6400-02B, Li-Cor Inc.) after 3 days of treatment (n = 10). Net photosynthetic rate (A_N_), stomatal conductance (g_s_), intercellular CO_2_ concentration (C_i_) and intrinsic water use efficiency (_i_WUE) were recorded under the following leaf chamber conditions: a photosynthetic photon flux density (PPFD) 1000 μmol photon m^−2^ s^−1^ (with 15% blue light to maximize stomatal aperture), vapour pressure deficit of 2.0–3.0 kPa and air, 50 ± 2% relative humidity, CO_2_ concentration surrounding leaf (C_a_) 400 and temperature of 13 ± 1 °C, 25 ± 1 °C and 40 ± 1 °C for plants grown at low, medium and high temperature ranges, respectively. Moreover, mesophyll conductance (g_m_) and maximum carboxylation rate allowed by ribulose-1,5-biphospate (RuBP) carboxylase/oxygenase (*V*_c,max_) were obtained by the curve-fitting method^44^ using the software package developed by Sharkey *et al*.^45^. For this method, four A_N_/C_i_ curves on randomly selected primary branches were performed. The curves were made under the same environmental conditions previously used for instantaneous gas exchange measurements. Once the steady-state was reached the curves were performed by decreasing C_a_ stepwise until 50 µmol mol^−1^. Therefore, to complete the curve the chamber conditions were restored to the initial and C_a_ increased stepwise until 2000 µmol mol^−1^ ^20,46^. For the curves, 12 different C_a_ values were used. At each step, gas exchange was allowed to equilibrate for less than 180 s to reduce changes in Rubisco activity^47^. On the same branches, dark respiration rate (R_d_, µmol CO_2_ m^−2^ s^−1^) measurements were performed after curves assessment as CO_2_ efflux. Branches chamber was darkened for 30 min to avoid transient post-illumination bursts of CO_2_ releasing^48^. Leakage of CO_2_ in and out of the leaf chamber was determined with photosynthetically inactive primary branches and corrected in all curves^48^. For all the measurements photosynthetic area was assimilated to the half the area of the cylindrical branches, since only the upper half received the unilateral illumination in the leaf chamber^49^.

Finally, the effect of the different salinity and maximum and minimum temperature ranges treatments on the limitations to A_N_ was tested, through to quantification of the absolute limitations of the observed decrease of A_N_ at the end of experiment period (3 d) by stomatal conductance (SL), mesophyll conductance (MCL) and biochemistry (BL) following the approach of Grassi and Magnani^50^.

### Measurement of chlorophyll fluorescence

Chlorophyll fluorescence measurements were performed using a FluorPen FP100 (Photo System Instruments, Czech Republic) in the same branches of gas exchange analysis at the end of the experiment (n = 7). Branches were dark-adapted for 30 min with special pliers designed for that purpose before the measurement. As Schreiber *et al*.^51^ described, light energy yields of Photosystem II (PSII) reaction centres were determined with a saturation pulse method. To estimate the maximum fluorescence signal across time, a saturating light pulse of 0.8 s with an intensity of 8000 µmol m^−2^ s^−1^ was used. A comparison of the minimum fluorescence (F’_0_), the maximum fluorescence (F’_m_) and the operational photochemical efficiency values were made with the values of dark adapted branches. Quantum yield of PS II (QY) were calculated as F_v_/F_m_.

Finally, the maximum electron transport rate (ETR_max_) and the chlorophyll *a* fast kinetics, or JIP-test (or Kautsky curves), which depicts the rate of reduction kinetics of various components of PSII, were also measured in dark-adapted leaves (n = 5 for each treatment) according to Duarte *et al*.^33^, using the pre-programmed RLC and OJIP protocols of the FluorPen. All derived parameters for both RLC and OJIP were calculated according to Marshall *et al*.^52^ and Strasseret *et al.*^29^ respectively. Furthermore, ETR_max_/A_N_ ratio was calculated with the values obtained from fluorescence rapid light curves and gas exchange measurements.

### Measurement of photosynthetic pigment concentration

At the end of the experimental period, branches samples were randomly collected (n = 5) and flash-frozen in liquid N_2_ and freeze-dried for 48 h in the dark to avoid photodegratation processes for the concentration analysis of photosynthetic pigments^31^. Samples were subsequently ground in pure acetone and pigments extracted at −20 °C during 24 h in the dark to prevent its degradation, centrifuged at 4000 rpm during 15 min at 4 °C and the resulting supernatant scanned in a dual beam spectrophotometer (Hitachi Ltd., Japan) from 350 to 750 nm at 1 nm step. The resulting absorbance spectrum was used for pigment quantification by introducing the absorbance spectrum in a Gauss-Peak Spectra (GPS) fitting library, using SigmaPlot Software^53^. De-epoxidation state index (DES) was calculated as:

$$([{\rm{Violaxanthin}}]+[{\rm{Anteroxanthin}}])/([{\rm{Violaxanthin}}]+[{\rm{Anteroxanthin}}]+[{\rm{Zeaxanthin}}])$$

### Measurement of anti-oxidant enzymatic activity

At the end of experiment, three replicates of 500 mg of fresh branches samples per treatment were ground in 8 ml of 50 mM sodium phosphate buffer (pH 7.6) with 0.1 mM Na-EDTA and were centrifuged at 10000 rpm for 20 min at 4 °C to obtaine the soluble proteins to determine anti-oxidant enzyme activities. Catalase (CAT) activity, was measured according to Teranishi *et al*.^54^, by monitoring the consumption of H_2_O_2_ and consequent decrease in absorbance at 240 nm (ε = 39.4 mM^−1^ cm^−1^). The reaction mixture contained 50 mM of sodium phosphate buffer (pH 7.6), 0.1 mM of Na-EDTA, and 100 mM of H_2_O_2_. Ascorbate peroxidase (APx) activity was measured by monitoring the decrease in the absorbance at 290 nm. The reaction mixture contained 50 mM of sodium phosphate buffer (pH 7.0), 12 mM of H_2_O_2_, 0.25 mM L-ascorbate^55^. Molar coefficient of 2.8 mM^−1^ cm^−1^ was used to calculate the amount of ascorbate oxidized. Guaiacol peroxidase (GPx) was calculated as Bergmeyer^56^ (1974) indicated. With a reaction mixture made of 50 mM of sodium phosphate buffer (pH 7.0), 2 mM of H_2_O_2_ and 20 mM of guaiacol. For all these enzymes activities the reaction was initiated with the addition of 100 µl of enzyme extract. To calculate the enzyme activity per µg of protein, total protein content in the extracts was obtained following Bradford^57^ (1976).

### Statistical analysis

All the statistic tests were performed by a statistical software package R. Two-way analysis of variance were used to analyze the interactive effect of each temperature ranges and NaCl concentrations (as categorical factors) treatments on the main physiological parameters (as dependent variables) of *S*. *ramosissima*. Multiple comparisons were analyzed by a Tukey (post hoc) test. The significance level considered to assume means differences was of P < 0.05. Before statistical analysis, Kolmogorov-Smirnov and Levene tests were used to verify the assumptions of normality and homogeneity of variances, respectively.
